# Effect of Surface Functionalization on the Cellular Uptake and Toxicity of Nanozeolite A

**DOI:** 10.1186/s11671-016-1334-8

**Published:** 2016-03-02

**Authors:** Sylwia Męczyńska-Wielgosz, Agata Piotrowska, Agnieszka Majkowska-Pilip, Aleksander Bilewicz, Marcin Kruszewski

**Affiliations:** Institute of Nuclear Chemistry and Technology, Dorodna 16, Warsaw, 03-195 Poland; Faculty of Medicine, University of Information Technology and Management in Rzeszów, ul. Sucharskiego 2, Rzeszów, 35-225 Poland; Department of Molecular Biology and Translational Research, Institute of Rural Health, Jaczewskiego 2, Lublin, 20-090 Poland

**Keywords:** Nanozeolite, PEG functionalization, Metabolic activity, Cytotoxicity

## Abstract

Extensive use of zeolite nanoparticles in many areas, including medicine, has led to the concern about an impact and possible risk of their use for human health and the environment.

In our studies, we investigated an uptake, retention, and cytotoxicity of nanozeolite A (BaA) functionalized with aminopropyl or poly(ethylene glycol) (PEG) of different chain lengths using human cervical carcinoma cell line. For internalization studies, nanozeolite was labeled with ^133^Ba radionuclide.

The results show that in the case of PEG modification, toxicity and uptake depend on the PEG chain length. The highest toxicity has been observed for nanozeolites coated with short-length chain (Ba-silane-PEGm(MW350). Also, amine-modified nanozeolites exhibited high toxicity, while nanozeolites coated with long PEG molecules, BaA-silane-PEGm(MW1000), and BaA-silane-PEGm(MW2000), as well as unmodified nanozeolite, seem to be nontoxic.

In conclusion, this study shows that uptake, retention, and toxicity of nanozeolites coated with various length PEG molecules groups depend on the molecular weight of PEG.

**Electronic supplementary material:**

The online version of this article (doi:10.1186/s11671-016-1334-8) contains supplementary material, which is available to authorized users.

## Background

Zeolites are biocompatible crystalline aluminosilicates composed of tetrahedral structures, which build open framework consisting of channels and cages of molecular dimensions. Each tetrahedron containing aluminum formally bears one unit of negative charge because aluminum atom is connected to four oxygen atoms. To make the crystal electrically neutral, metal cations (e.g., Na^+^, K^+^, Ca^2+^) are present in the interstices of the zeolitic framework. Zeolites were first discovered at the end of the eighteenth century and came to broad application about 50 years ago, when synthetically obtained zeolites were used in many branches of industry, as molecular sieves for gas adsorption and purification processes or as catalysts in the petroleum industry. Up to data, 194 different zeolite structures with various physicochemical properties are known. Nowadays, with reduction of the particle size from the micrometer to the nanometer scale, nanozeolites turned out to be a very flexible material with increasing use in selective catalysis [1], as components of mixed matrix composite membranes [2, 3], biosensors [4, 5], agents for cancer diagnosis [6–8], drug delivery carrier [9, 10], and in separation of organics and ions in liquid processing [11]. Due to their high porosity and negatively charged interior, nanozeolites are an effective cage for cations or positively charged small molecules that can be trapped in nanozeolites’ channels without any chemical modifications. For instance, Gd^3+^-doped NaA or NaY nanozeolites are the potential MRI contrast agent [12, 13]. Also, it was shown that nanometer zeolite L can be functionalized, using different groups, leading to a dual probe contrast agent for MRI and optical imaging [14].

Recent study indicates that size, area, solubility, shape, surface area, and chemistry have an impact on toxicity of nanoparticles (NPs) [15]. Also, surface characteristics of NPs are a very important property, especially for medical application. The surface of nanozeolites can be functionalized with different coupling agents that gives the possibility of binding different molecules. By changing NP surface properties, researchers can affect their biocompatibility and fate in the body. When in contact with biological fluids or cellular content, bare NPs are rapidly covered by biological macromolecules, e.g., proteins, lipids, and carbohydrates, that change their biological properties. For example, when injected directly to the blood, the serum proteins act like opsonins to entail elimination of injected NPs from the circulation [16]. If quick cleaners are not advantageous, NPs can be coated with polymer materials, such as dextran, chitosan, poly(vinyl alcohol), poly(ε-caprolactone), or poly(ethylene glycol) (PEG) [17, 18]. Those hydrophilic polymers bound to NP surface diminish the opsonization process that results in longer half-life of coated nanoparticles in the bloodstream. Moreover, modification of NP surface with polymers improves their stabilization in water and reduces particle agglomeration [19]. The most commonly used polymer is PEG. The high solubility, both in aqueous and organic media, makes PEG and it derivatives very suitable agents for molecule coupling in a variety of conditions [20].

There are many PEG derivatives commercially available. Among them, PEG-silanes are the most often used to modify nanozeolites’ surface. The functionalization route is known as silanization, and several silanization methods have been developed so far [21, 22]. Tsotsalas et al. [6] used organic solvent to covalently attach PEG-silane molecules to hydroxyl groups on the nanozeolite L surface to make it more hydrophilic. However, subsequent studies showed no effect of modification on NP biodistribution, which authors attributed to the too short chain length of the chosen PEG molecule.

Increased application of nanozeolites in many areas led to the concern about their impact and possible risk for human health and the environment. The aim of this study was to examine the impact of nanozeolite surface modification on its cellular uptake, retention inside the cell, and cytotoxicity in vitro. The study was performed using nanozeolite A (loaded with Ba^2+^ and spiked with ^133^Ba) functionalized with PEG of different chain lengths on human cervical carcinoma cell line (HeLa).

## Methods

### Test Materials and Reagents

If not indicated otherwise, all reagents were from Sigma-Aldrich (Poland). The following chemical reagents were used: Ludox CL-X colloidal silica 45 % suspension in water, sodium aluminate, sodium hydroxide (pure p.a., Chempur, Poland), tetramethylammonium hydroxide (TMAOH 25 % wt.), ethanol (99.9 %, POCH, Poland), acetic acid (POCH, Poland), methoxyl-silane-PEG (silane-PEGm; MW350, MW1000, and MW2000), and (3-aminopropyl)triethoxysilane (silane-NH_2_, ≥98 %). For biological studies, the following materials were used: RPMI-1640, L-glutamine, phosphate-buffered saline (PBS), dimethylsulfoxide (DMSO), 3-(4,5-dimetyl-2-thiazolyl)-2,5-diphenyl-2H-tetrazolium bromide (MTT), ethanol, and neutral red (NR). EMEM media was a product of the American Type Tissue Culture Collection (ATCC, Rockville, MD). Fetal calf serum was a product of Biological Industries (Israel). ^133^Ba radionuclide was purchased from POLATOM (Poland).

### Cell Culture

HeLa cells and human embryonic kidney 293 cells (HEK-293) were purchased from the American Type Tissue Culture Collection (ATCC, Rockville, MD) and maintained according to the ATCC protocol. Briefly, HeLa were cultured in RPMI-1640 medium supplemented with 10 % fetal calf serum (FCS) and 2 mM glutamine. HEK-293 was cultured in EMEM medium supplemented with 10 % FCS. The cells were incubated in a 5 % CO_2_ atmosphere at 37 °C.

HeLa cells are one of the best characterized human cell lines ever and one of the most popular cellular models in science and industry [23]. Over 50 years, the cells are used in biomedical studies and recently in nanotoxicology and nanomedicine, including the nanoparticles uptake studies [24].

### Synthesis of NaA Nanozeolite and its Surface Modification with Silane Coupling Agents

The sodium form of an A type of nanozeolite (NaA) was synthesized using the method recently reported [9]. In brief, sodium aluminate was added to an aqueous sodium hydroxide solution and kept under stirring to complete dissolution. Then, the required amount of tetramethylammonium hydroxide (template) was slowly added and the solution and stirred continuously for 24 h at room temperature. In the next step, a colloidal silica was added dropwise to the solution under stirring and the mixture was immediately placed in an oil bath equipped with a magnetic stirrer and temperature controller. Hydrothermal crystallization was carried out for 48 h, at 100 °C with rotation rate 750 rpm. The obtained nanocrystals were cooled to room temperature and washed with distilled water, spun down at 13,500 rpm (2-16P Spincontrol Universal, Germany) until the supernatant pH value was below 8. Next, the sample was calcinated at 500 °C for 3 h to remove the template.

In order to functionalize the NaA nanozeolite surface, the ethanol solution containing 4 % water (*v*/*v*) was prepared and its pH was adjusted to 4.5–5.5 with acetic acid. Ten milligrams of silane-PEGm (MW350) was added to the 20 mL of acidic water/ethanol solution under stirring at room temperature. The reagents were stirred continuously for 5 min. After that, 10 mg of nanozeolite NaA was added to the solution and stirring was continued for 1 h, at room temperature. The obtained product was washed several times with ethanol to remove excess silane-PEG compound and dried at 110 °C for 30 min. The same procedure was carried out for silane-PEGm (MW 1000), silane-PEGm (MW 2000), and silane-NH_2_ coating.

### Labeling Nanozeolites with ^133^Ba^2+^ and Ba^2+^

Prior to the internalization experiment, Na^+^ ions present in the nanozeolite structure were exchanged for ^133^Ba^2+^ (or Ba^2+^) ions by a simple ion-exchange process:

$$ {{2\mathrm{N}\mathrm{a}}_{\mathrm{z}}}^{+}{+}^{133}{{\mathrm{Ba}}_{\mathrm{s}}}^{2+}\leftrightarrow\ {{2\mathrm{N}\mathrm{a}}_{\mathrm{s}}}^{+}{+}^{133}{{\mathrm{Ba}}_{\mathrm{z}}}^{2+}, $$

where z index means ions in the zeolite structure and s index means ions in the solution, in which nanozeolite sample was suspended. The ion-exchange process was conducted according to the method described below. Ten milligrams of NaA nanozeolite was suspended in 2 mL of ^133^BaCl_2_ solution (*A* = 5 kBq), POLATOM, Poland) or BaCl_2_ solution in an Eppendorf tube and sonicated in an ultrasound bath for 15 min. Then, the Eppendorf tube was gently shaken on a circular stirrer for 2 h and the suspension was centrifuged for 15 min at 13,000 rpm (MiniSpin, Eppendorf, Poland). The precipitate was washed several times with deionized water, resuspended in 1 mL of deionized water, and sonicated.

Presence of radioactive tracer ^133^Ba inside the nanozeolite was crucial for radiometric detection of NaA during internalization studies. It is well known that the A type of nanozeolite is very selective for Ba^2+^ ions, which assured getting the stable barium ions bound inside nanozeolite A. As the presence of radioactive barium ions might obscure the results of cytotoxicity studies, to provide that each sample will be chemically identical with these used in internalization studies, Na^+^ ions were exchanged with non-radioactive Ba^2+^ ions.

### Transmission Electron Microscopy

The particle size and morphology of the synthesized A-type nanozeolite was determined by transmission electron microscopy (TEM, LEO 912AB). Suspension of nanozeolites in ethanol was laid on a copper gird and left to dry in air.

### Dynamic Light Scattering

The average size and zeta potential (ζ) were measured by dynamic light scattering (DLS, Malvern, UK). Both the hydrodynamic diameter and zeta potential of samples were measured at 25 °C with a scattering angle of 173°. The particle concentration for hydrodynamic diameter measurements was 30 μg/mL in 0.001 M phosphate buffer (pH 7.4), and the measurement was done in triplicate with over 17 sub-runs. The particle concentration for zeta potential measurements was 330 μg/mL in 0.001 M phosphate buffer (pH 7.4), and the measurement was done in triplicate with over 13 sub-runs.

### Thermogravimetric Analysis

Thermal stability of PEG-coated nanozeolites and the PEG molecules content per nanoparticle was determined by thermogravimetric analysis (TGA, Q500, TA Instruments, USA). About 5 mg of dried substance was placed in a TG furnace, and the analysis was conducted from 20 to 800 °C at a heating rate of 10 °C per min, under nitrogen.

### Radioactivity Measurement

The radioactivity of fractions collected during internalization and priming experiments was measured using Wizard ^2^® automatic gamma counter 2480 (Perkin Elmer) equipped with a thallium-activated sodium iodide crystal NaI(Tl) detector. The crystal height is 80 mm and diameter is 75 mm.

### Cytotoxicity Evaluation

The impact of modified nanozeolites on metabolic activity and proliferation of HeLa and HEK-293 cells was measured with MTT and neutral red (NR) assay, respectively. The assays were performed as described in [25]. In brief, cells were seeded in 96-well microplates (TPP, Switzerland) at a density of 1 × 10^4^ cells/well in 100 μL of culture medium. At least three independent experiments in six replicate wells were conducted per concentration. Twenty four hours after cell seeding, cells were treated with increasing concentrations of modified nanozeolites (5, 10, 25, 50 μg/mL) (1.5, 3, 7.5, 15 μg/cm^2^) for 24, 48, and 72 h. After treatment cell culture medium was removed, the cells were washed with 150 μL PBS. For MTT assay, 100 μL of 3 mg/mL MTT solution was added to each well. After 3 h incubation at 37 °C, the MTT solution was removed. Remaining insoluble formazan crystals were dissolved in 100 μL DMSO, and absorbance was measured at 570 nm in plate reader spectrophotometer Infinite M200 (Tecan, Austria). For NR assay after treatment, cells were incubated for 3 h at 37 °C with 100 μL of neutral red solution (stock solution of NR 5 mg/mL in PBS was diluted 1:100 in cell culture medium, incubated for 12 h in 37 °C, and centrifuged to remove any undissolved NR powder). Next, NR solution was aspirated, cells were washed with 150 μL of PBS, and 200 μL of an acid-ethanol solution (49 % water, 50 % ethanol, and 1 % acetic acid) was added to each well. After 15 min of gentle shaking, optical density was read at 540 nm in plate reader spectrophotometer Infinite M200 (Tecan, Austria).

### BaA Internalization

The cells were seeded into 6-well plates (TPP, Israel) at 5 × 10^5^ cells per well and cultured for 24 h. Then, cells were incubated at 37 °C for 1, 3, 6, or 24 h with 50 μg/mL (15 μg/cm^2^) of [^133^Ba]BaA, [^133^Ba]BaA-silane-PEGm(MW350), [^133^Ba]BaA-silane-PEGm(MW1000), [^133^Ba]BaA-silane-PEGm(MW2000), or [^133^Ba]BaA-silane-NH_2_. After treatment, growth medium of each well was collected; the cells were washed twice with 750 μL of PBS and incubated with 500 μL of pre-warmed trypsin solution at 37 °C for 10 min. Subsequently, the cells were collected and activity of each fraction was measured. The results are expressed as a percentage of the total radioactivity added.

### BaA Priming

The cells were seeded into 6-well plates (TPP, Israel) at 5 × 10^5^ cells per well and cultured for 24 h. Then, cells were treated for 1 h at 37 °C with 50 μg/mL (15 μg/cm^2^) of [^133^Ba]BaA, [^133^Ba]BaA-silane-PEGm (MW350), [^133^Ba]BaA-silane-PEGm(MW1000), [^133^Ba]BaA-silane-PEGm(MW2000), or [^133^Ba]BaA-silane-NH_2_. After incubation, medium was removed from each well, the cells were washed twice with 750 μL of PBS, and the fresh medium was added to the cells. Next, the cells were incubated at 37 °C for 3, 6, or 24 h. After incubation, medium of each well was collected; cells were washed twice with 750 μL of PBS and incubated with 500 μL of pre-warmed trypsin solution at 37 °C for 10 min and collected. The activity of each fraction was measured. The results are expressed as a percentage of the total radioactivity internalized after 1 h of incubation.

### Statistical Analysis

At least three independent experiments were conducted for each toxicity and internalization point. Difference between samples and control were evaluated using GraphPad Prism 5.0 software (GraphPad Software Inc., USA). Internalization and priming data were analyzed by pairwise comparison by Student *t* test for independent and dependent samples, respectively. Toxicological data were evaluated by Kruskal-Wallis One Way Analysis of Variance on Ranks (ANOVA) followed by post hoc Dunnet’s method. Differences were considered statistically significant when the *p* value was <0.05.

## Results

### Nanoparticles Characterization

**Fig. 1:**
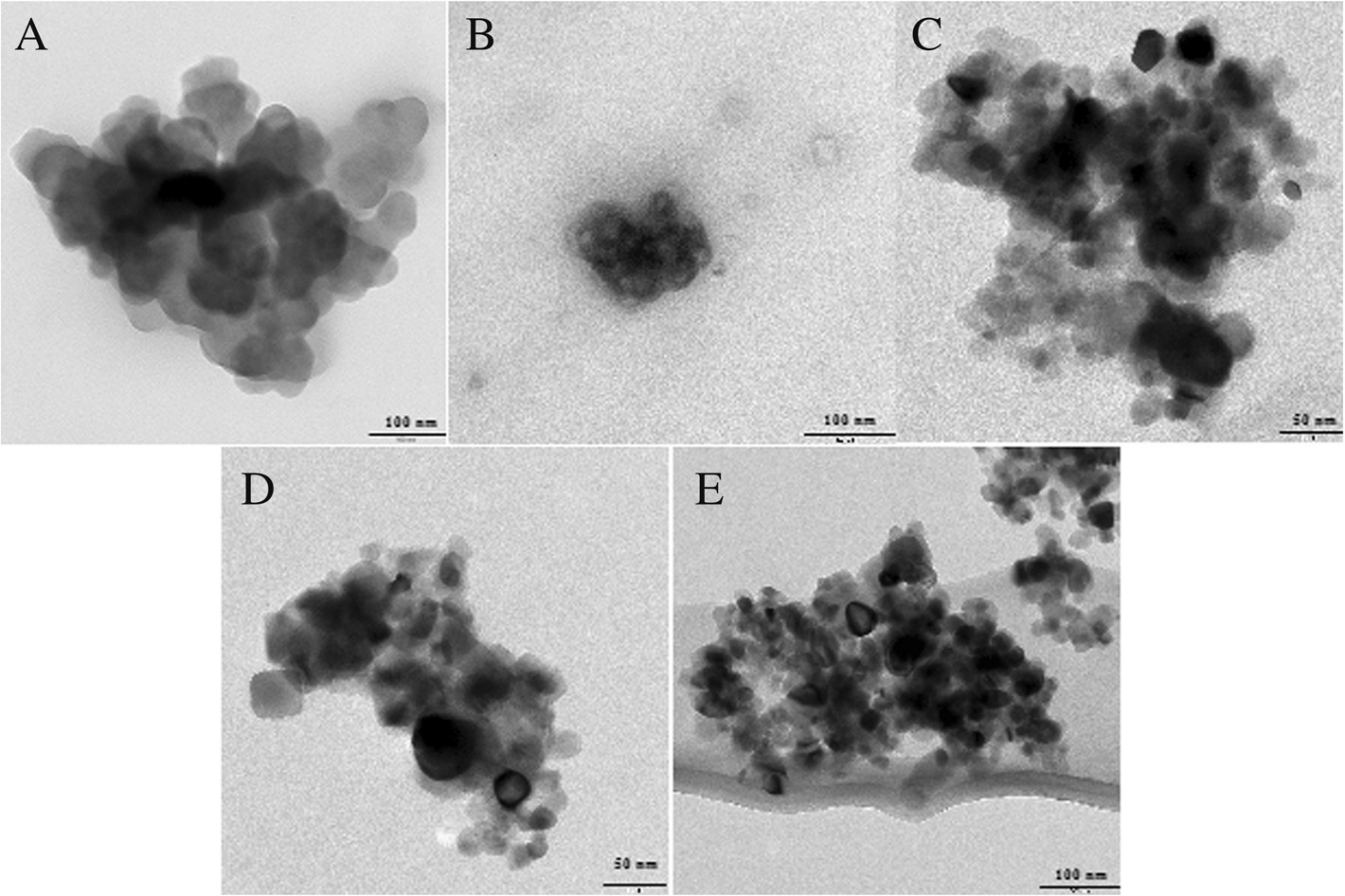
TEM images of **a** BaA, **b** BaA-silane-PEGm (MW350), **c** BaA-silane-PEGm (MW1000), **d** BaA-silane-PEGm (MW2000), and **e** BaA-silane-NH_2_

Figure 1 shows the transmission electron microscopy (TEM) images of modified and unmodified nanozeolite BaA samples. The estimated average diameter of all obtained nanozeolites was 50–60 nm, as measured on the basis of TEM images. TEM microphotographs showed also that the morphology of all nanozeolites was similar which is crucial for the investigation of the surface chemistry dependent cytotoxicity.

Size distribution and zeta potential of synthesized nanozeolites in water are summarized in Table 1. Nanozeolite coating with short-length PEG molecules (BaA-silane-PEG MW350) and silane terminated with –NH_2_ groups (BaA-silane-NH_2_-PEG of NW 350) resulted in dramatic increase in hydrodynamic diameter, whereas coating with PEG MW 1000 and 2000 did not markedly increased the NP hydrodynamic diameter. Despite the differences in hydrodynamic diameter, zeta potential of functionalized nanozeolites systematically increased with the increase of the length of PEG molecule coupled to the NP surface. The lowest value of zeta potential was observed for the bare BaA nanozeolites, whereas the highest one for the amino group-modified ones (Table 1).

**Table 1:** Hydrodynamic diameter and zeta potential of functionalized nanozeolite BaA NPs in water

	Average hydrodynamic diameter (nm)	Polydispersity index (PDI)	Zeta potential (mV)
BaA	159.5 ± 3.4	0.248 ± 0.02	−75.2 ± −0.6
BaA-silane-PEGm (MW350)	255.5 ± 15.1	0.256 ± 0.07	−65.6 ± −2.5
BaA-silane-PEGm (MW1000)	158.2 ± 4.6	0.289 ± 0.03	−50.1 ± −1.1
BaA-silane-PEGm (MW2000)	175.2 ± 11.8	0.251 ± 0.02	16.0 ± 0.2
BaA-silane-NH_2_	254.3 ± 12.4	0.276 ± 0.05	30.4 ± 1.1

Data are expressed as mean of three experiments ± SD

To confirm the surface modification and to estimate the number of modifying molecules on the NP surface, the thermogravimetric (TGA) analysis was performed (Additional file 1: Figure S1). The mass loss peak observed at around 100 °C is attributed to the dehydration of the sample. However, at 390 °C, the second mass loss of about 2–3 % is observed for each modified nanozeolite. This is attributed to the breaking of silane–nanozeolite bond and liberation of the silane-coupled molecules. On the base of obtained TGA results, the number of silane-PEG and silane-NH_2_ molecules per one NP can be estimated. The calculation was performed under assumption that NPs were spherical with a diameter of 60 nm and density of material was 2.5 g/cm^−3^. As expected, the number of functionalizing molecules changed according to the size of molecule. The highest number of attached molecules was revealed for low-mass NH_2_ and PEG(MW350) molecules. Whereas no big difference was observed for PEG(MW1000) and PEG(MW2000) molecules (Table 2).

**Table 2:** Quantity of functionalizing molecules attached to the one zeolite A NP

	The number of functionalizing molecules attached to the one zeolite A NP
BaA-silane-PEGm (MW350)	5500
BaA-silane-PEGm (MW1000)	2400
BaA-silane-PEGm (MW2000)	2100
BaA-silane-NH_2_	9500

### Cytotoxicity

The cytotoxic activity of unmodified and modified nanozeolites BaA was investigated on HeLa and HEK-293 cells by two independent methods, namely the MTT and neutral red assays. These two assays are based on different principles and were used to eliminate a possible interference of tested nanozeolites with the assay [26, 27]. Nevertheless, as interference of nanoparticles with tetrazolium-based assays is widely recognized, before starting the experiments, the credibility of the MTT assay in regards to nanozeolite was tested in a cell-free system. No interference of nanozeolites with the MTT assay was observed in the concentrations range used in the further cytotoxicity study (Additional file 2: Figure S2, Additional file 3).

**Fig. 2:**
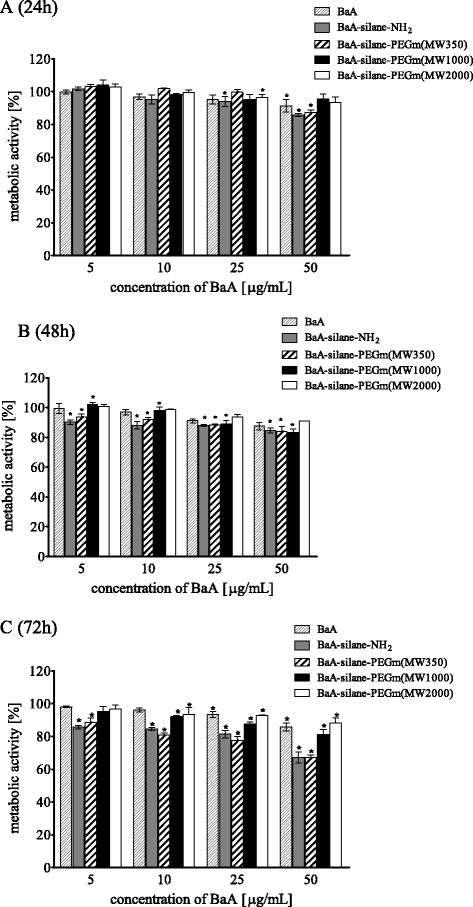
Metabolic activity (MTT assay) of HeLa cells treated with different concentrations of unmodified and modified BaA nanozeolites for 24 h (**a**), 48 h (**b**), or 72 h (**c**). Data are expressed as a percent of control, mean ± SD from three independent experiments (*asterisk* denotes statistically significant difference from unexposed control, *p* < 0.05)

**Fig. 3:**
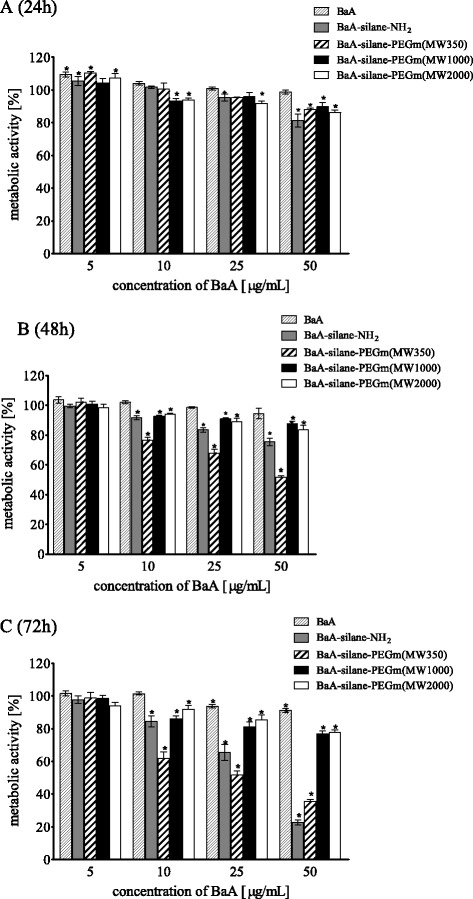
Metabolic activity (MTT assay) of HEK-293 cells treated with different concentrations of unmodified and modified BaA nanozeolites for 24 h (**a**), 48 h (**b**), or 72 h (**c**). Data are expressed as a percent of control, mean ± SD from three independent experiments (*asterisk* denotes statistically significant difference from unexposed control, *p* < 0.05)

As shown in the Fig. 2 metabolic activity (MTT assay) of HeLa cells exposed to nanozeolites, BaA modified with different molecular weight PEGs varied significantly. BaA, BaA-silane-PEGm(MW1000), and BaA-silane-PEGm(MW2000) were relatively nontoxic; a significantly decreased mitochondrial activity was observed only at the highest dose (50 μg/mL) (15 μg/cm^2^). This concentration reduced the metabolic activity of HeLa cells after 72 h incubation to 85, 87, and 80 % of control value, for BaA, BaA-silane-PEGm(MW2000), and BaA-silane-PEGm(MW1000), respectively. In contrast, treatment of the cells with BaA-silane-PEGm(MW350) or BaA-silane-NH_2_ resulted in a significant reduction of metabolic activity in time-dependent manner. The highest concentration of both, BaA-silane-PEGm(MW350) and BaA-silane-NH_2_, reduced the metabolic activity of HeLa cells after 72 h incubation to 67 %. The results of experiments with HEK-293 cells showed a similar tendency as in the case of the HeLa cells. BaA, BaA-silane-PEGm(MW1000), and BaA-silane-PEGm(MW2000) were relatively nontoxic; a significantly decreased mitochondrial activity was observed only at the highest dose (50 μg/mL or 15 μg/cm^2^). This concentration reduced the metabolic activity of HEK-293 cells after 72 h incubation to 91 %, 76 % and 77 % of control value for BaA, BaA-silane-PEGm(MW2000) and BaA-silane-PEGm(MW1000), respectively. Treatment of the cells with BaA-silane-PEGm(MW350) and BaA-silane-NH_2_ reduced significantly the cells’ metabolic activity in time and dose-dependent manner. The highest concentration of BaA-silane-PEGm(MW350) and BaA-silane-NH_2_ reduced the metabolic activity of HEK-293 cells after 72 h incubation to 22 % and 35 % of control value for Ba-silane-PEGm(MW350) and BaA-silane-NH_2_ respectively (Fig. 3).

**Fig. 4:**
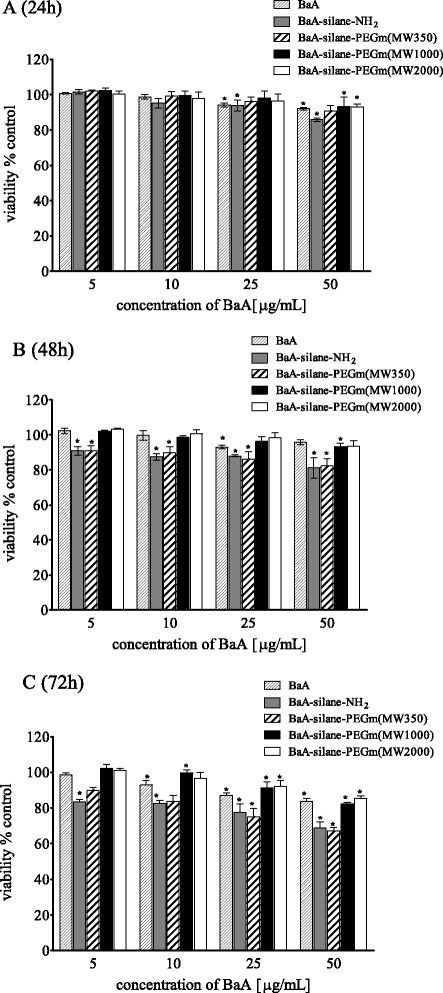
Viability (NR assay) of HeLa cells treated with different concentrations of unmodified and modified BaA nanozeolites for 24 h (**a**), 48 h (**b**), or 72 h (**c**). Data are expressed as a percent of control, mean ± SD from three independent experiments (*asterisk* denotes statistically significant difference from unexposed control, *p* < 0.05)

**Fig. 5:**
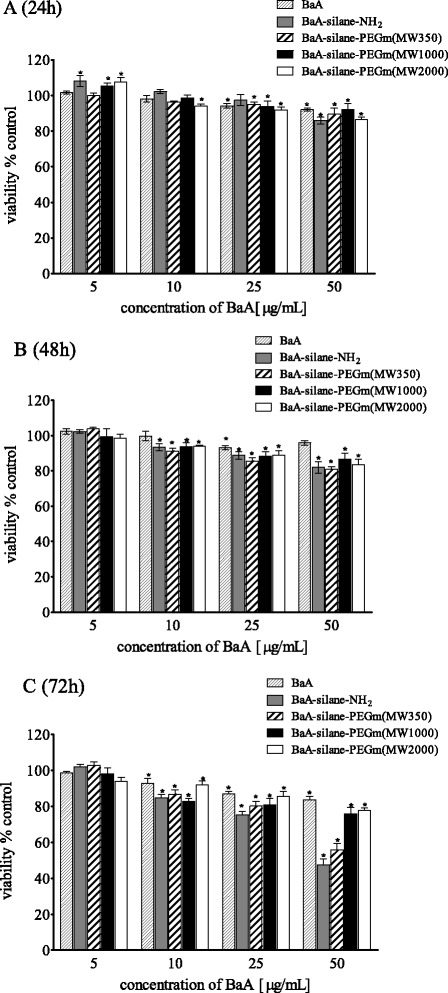
Viability (NR assay) of HEK-293 cells treated with different concentrations of unmodified and modified BaA nanozeolites for 24 h (**a**), 48 h (**b**), or 72 h (**c**). Data are expressed as a percent of control, mean ± SD from three independent experiments (*asterisk* denotes statistically significant difference from unexposed control, *p* < 0.05)

Cell viability estimated by neutral red assay shows a similar trend as MTT assay. For both cell lines, nanozeolite BaA, BaA-silane-PEGm(MW1000), and BaA-silane-PEGm(MW2000) showed low toxicity, slightly increasing with the concentration. For these compounds, a statistically significant decrease in viability was observed only at 50 μg/mL (15 μg/cm^2^) after 72 h treatment. On the contrary, BaA-silane-PEGm(MW350) or BaA-silane-NH_2_ caused significant decrease of viability in a dose- and time-dependent manner. The highest used concentration reduced the viability of HeLa cells after 72 h incubation to 65 and 64 %, for BaA-silane-PEGm(MW350) and BaA-silane-NH_2_, respectively (Fig. 4). For HEK-293 cells, the highest used dose reduced the viability of cells after 72 h incubation to 48 % for BaA-silane-PEGm(MW350) and to 55 % for BaA-silane-NH_2_ (Fig. 5).

Taken together, these results show that nanozeolites BaA, BaA-silane-PEGm(MW1000), and BaA-silane-PEGm(MW2000) are relatively nontoxic, whereas the impact on both cells of BaA-silane-PEGm(MW350) and BaA-silane-NH_2_ is more pronounced.

### Nanozeolites Internalization and Turnover

**Fig. 6:**
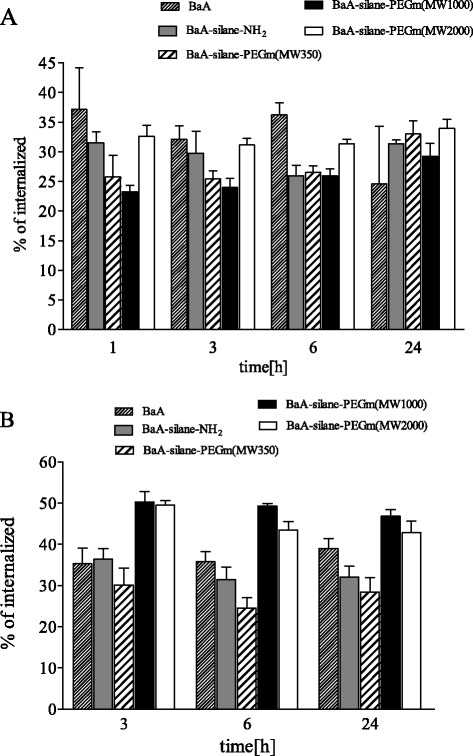
Internalization (**a**) and retention (**b**) of unmodified and modified nanozeolites BaA in HeLa cells. Nanozeolites are ranked according to the size of coating molecule. Data are expressed as a mean ± SD from three independent experiments. Statistical analysis of the data is presented in the Additional files 4: Table S1 and 5: Table S2

HeLa cells were incubated with 50 μg/mL (15 μg/cm^2^) of ^133^Ba radiolabeled nanozeolites, and their uptake was assayed up to 24 h (Fig. 6a). The highest percent of internalized radioactivity was observed for the bare [^133^Ba]BaA, [^133^Ba]BaA-silane-PEGm(MW2000), and [^133^Ba]BaA-silane-NH_2,_ and resulted in more than 37, 33, and 32 % of added radioactivity after 1 h incubation, respectively. The value of internalized radioactivity decreased after 24 h to 25 % for [^133^Ba]BaA, but for [^133^Ba]BaA-silane-PEGm(MW2000) and [^133^Ba]BaA-silane-NH_2_ was constant in this period. The lower amount of internalized radioactivity has been observed for [^133^Ba]BaA-silane-PEGm(MW1000) and [^133^Ba]BaA-silane-PEGm(MW350), and yielded about 22 and 25 % after 1 h of incubation, respectively. Interestingly, being relatively constant till 6 h incubation, the radioactivity of internalized [^133^Ba]BaA-silane-PEGm(MW1000) and [^133^Ba]BaA-silane-PEGm(MW350) significantly increased after 24 h incubation (to 28 and 34 %, respectively).

The intracellular retention of nanozeolites BaA was assessed by priming experiment. HeLa cells were incubated with 50 μg/mL (15 μg/cm^2^) of radiolabeled nanozeolites for 1 h; then, the radioactive medium was exchanged with a non-radioactive one and released radioactivity was measured after 2, 5, and 23 h (the total incubation time was 3, 6, and 24 h, respectively). The results obtained are presented on Fig. 6b. A majority of internalized radioactivity was lost during the first 3 h of post-incubation. The biggest loss was observed for the BaA-silane-PEGm(MW350) nanozeolite (70 %), whereas the smallest for BaA-silane-PEGm(MW1000) and BaA-silane-PEGm(MW2000) nanozeolites (50 %). The amount of remaining intracellular radioactivity was relatively stable till 24 h, when the experiments ended. Only slight decrease of intracellular radioactivity was observed in the samples treated with BaA-silane-PEGm(MW1000) and BaA-silane-PEGm(MW2000) nanozeolites.

## Discussion

In the present work, we prepared nanozeolite A of the same size and shape, but differing coating molecules, to investigate the impact of surface properties on the nanozeolite toxicity and its interaction with mammalian cells. The surface of BaA nanozeolite was modified with four different coatings molecules, a silane terminated with the –NH_2_ group and silane terminated with different chain length PEG molecules (the molecular weight of PEG was 350, 1000, and 2000). Due to formation of hydration layer, size of unmodified BaA nanozeolite determined by DLS was much larger than this observed on TEM images, which is in good agreement with data reported in literature [28]. Coating of NPs with PEG of MW1000 and MW2000 did not resulted in significant increase of hydrodynamic diameter of the NP, but coating with PEG of MW350 resulted in dramatic increase of hydrodynamic diameter. This might be associated with different grafting density of PEG 350 and PEG1000 or PEG2000 (Table 2). The dense grafting of PEG350 molecules may force the brush conformation of the PEG molecule, whereas for less dense packaged PEG1000 or PEG2000 mushroom conformation should be rather expected [29]. This in turn resulted in difference in hydrodynamic diameter of NP. Indeed, the increase of density of PEG5000 molecules grafted on gold NP was accompanied by the systematic increase of NP hydrodynamic diameter [30]. Moreover, although gradual increase of NP hydrodynamic diameter according to the PEG molecule chain length was observed for high molecular weight PEG molecules, only small increase of the parameter was reported for PEG2000 molecules [31, 32].

The bare BaA nanozeolite possessed very negative surface charge, higher than other nanozeolites, which was explained by the high amount of aluminum content in the framework of this type of structure. Functionalization of the nanozeolite surface with increasing length PEG molecules systematically decreased negative charge of the NPs. Finally, it became positive for the longest one (PEG MW2000). The increase of NP zeta potential with the increase of grafted PEG molecules length or number was also observed by the others for different types of NPs, including metallic and non-metallic ones [31–33]. Thus, the decrease of zeta potential of PEG-grafted BaA is another proof for its successful PEGylation. The presence of PEG on the surface moves the slipping plane further away from the NP surface [34] and/or the drag caused by the presence of the PEG chains on the NP surface reduces its mobility (and hence the zeta potential). Also, attachment of the amino group to the nanozeolite surface drastically changed the NP zeta potential, which rises to a positive +30 mV value. The positive charge of BaA-silan-NH_2_ NP surface is likely due to the protonation of amine groups.

The surface charge affects NP interaction with the cells [35]. The surface of the cell membrane is negatively charged; thus, theoretically, positively charged nanoparticles should be absorbed much easier than negatively charged once. Hence, positively charged NPs should show higher level of internalization. The better uptake of positively charged NPs was observed for gold and silver, superparamagnetic iron oxide, hydroxylapatite, silicon dioxide, lipid, poly(lactic acid), chitosan, polymeric particles, and polystyrene NPs, as compared to the respective anionic ones [25, 36–44].

In our experiments, two positively charged nanozeolites, namely BaA-silane-PEGm(MW2000) and BaA-silane-NH_2_, showed a very high level of internalization, which is in a good agreement with the above-cited literature. Interestingly, the most negatively charged NPs (bare BA NPs) also showed a very high level of internalization up to 6 h, then tended to decrease. This observation might point to different cellular mechanisms responsible for the uptake of positively and negatively charged NPs. Indeed, high uptake of negatively charged NPs was observed for phagocytic cells, such as macrophages and monocytes, presumably due to the ingestion of bacteria, which also display a net negative charge [45]. Although phagocytic capabilities of the HeLa cells are much lower than macrophages, it cannot be ruled out that similar mechanisms might be present in this case, however, to the lesser extent. Thus, only highly negative NPs would be uptaken, whereas those with the surface charge close to neutral do not interact with the cellular membrane. This is supported by the observation of Patil et al. [46], who examined internalization of cerium oxide nanoparticles and found that negatively charged nanoparticles more efficiently internalized into adenocarcinoma lung cells than the positively charged.

The priming studies showed that the turnover of nanozeolites is rather slow, and a large portion of NPs internalized during the first hour of incubation retained in cells up to 24 h afterwards. Nanozeolites with the longest PEG chain (PEG MW 1000 and 2000) showed the highest retention, despite the fact that one was positively charged (BaA-silane-PEGm(MW2000)), whereas the other negatively charged (BaA-silane-PEGm(MW1000)). This suggest that, in contrast to internalization, intracellular trafficking and NP excretion depend on the surface properties other than the surface charge.

Just few reports regarding potential toxicity of nanozeolites are available till now. The first one concerns the in vitro cytotoxic response of macrophages, epithelial and endothelial cells to nanozeolites A and Y (25–100 nm) [47]. The study showed very low toxicity of nanozeolites. No toxicity was also reported for pure silica MFI zeolite 50 and 100 nm nanoparticles [48] and LTL zeolite 80 nm nanoparticles [49]. Another group investigated the influence of size, composition, and morphology of nanozeolite on its in vitro cytotoxicity. From the more than 200 different framework types, the authors selected those varied in aluminum content, such as LTL, LTA, MFI, and ZSM-1 and additionally a pure silica nanozeolite (silicalite-1). The results revealed that toxicity is relative to the nanozeolite structure and chemical composition. The pure silica nanozeolites of spherical shape were nontoxic, whereas toxicity of aluminum containing nanozeolites depend on the shape and dose. The authors suggested that the surface of aluminum containing nanozeolites should be modified to decrease the toxicity and allow their future medical application [50].

In our hands, the toxicity of nanozeolites showed a dose- and time-dependent mode, both in normal (HEK-239) and cancer (HeLa) cells. The cancer cells proved to be less susceptible to the toxic action of nanozeolite than normal cells. However, this should be expected due to the constitutive activation or increase of many anti-apoptotic and/or pro-survival signaling pathways in cancer cells [51]. The coating of nanozeolites differently influenced their toxicity, and both the length of PEG chain and type of modified group (in particular –NH_2_) had significant impact on their toxicity. As the size and shape of each nanozeolite is the same, the toxicity must be strictly connected with the type of the surface coating molecules. It is known that PEG coating decreases the NP aggregation, uptake, and also affects theirs toxicity. Xie et al. [52] showed that PEG coating on Fe_3_O_4_ NPs protect the NPs from aggregation in cell culture medium and decreased nonspecific uptake by macrophages. This uptake was dependent on the length of the PEG chain and decreased with the increase of PEG molecular weight. There are also many researches showing that toxicity of nanoparticles coated with PEG is smaller than those of bare nanoparticles [53]. Luo et al. [54] showed that amine group-modified bismuth NPs were the most toxic, whereas PEG-modified ones expressed the lowest toxicity due to the low zeta potential and reduced interaction with receptors. Moreover, PEG-modified bismuth NPs showed weak affinity to proteins present on the cell surface, and therefore, most of Bi-PEG nanoparticles are expelled outside the cell membrane. Interestingly, PEG-coated silver nanoparticles were more toxic than Ag ions at similar concentration, despite the fact that nanoparticles coated with 5 kDa PEG were more toxic than those coated with 20 kDa [55].

In addition, different studies have shown that nanoparticle hydrophilicity increases with increasing length of PEG chain bounded to the particle surface. Hydrophilic surface may prevent nanoparticles suspended in aqueous solution from aggregation [53]. Our results obtained by DLS seem to support this observation (Table 1). Nanoparticles BaA-silane-PEGm(MW350) and BaA-silane-NH_2_ seem to be less stable and tend to produce particles with hydrodynamic diameter about 100 nm higher than BaA-silane-PEGm(MW2000), BaA-silane-PEGm(MW1000), and BaA nanozeolites, which might be associated with the formation of small particles agglomerates.

## Conclusions

It is commonly accepted that toxicity of NPs depends on their chemical composition, size, shape, and surface properties. In the present work, we used same type, size, and shape nanozeolites to examine the impact of the surface modification on their cytotoxicity on HeLa cells. Whereas our results confirm the general assumption, they also point to the role of surface charge in NP cellular turnover. Our results show also that toxicity of nanozeolites coated with various-length PEG molecules depends on the molecular weight of PEG. Although the exact mechanism by which PEG molecules modify NP toxicity is still unclear, at least two possible means come to mind: (1) changing of the NP surface properties, such as hydrophilicity, that in turn modifies NP aggregation rate and its bioavailability, and (2) modification of cell-NP interaction due to the different NP’s surface structure. As conformation of PEG molecules on the NP surface depends on their length, it cannot be ruled out that particular PEG molecules conformation is advantageous for NP interaction with cell membrane and its internalization.

PEG is commonly used for NP surface derivatization to make NPs more biocompatible; however, our results indicate that the concerted influence of the PEG molecule length and NP surface charge determine the NP uptake, turnover, and toxicity. Thus, for the purpose of medical application, to diminish NP toxicity, the most suitable coating for nanozeolites is PEG with long chain (e.g., MW 1000 or 2000).

## Additional files

Additional file 1: Figure S1. TGA analysis of modified nanozeolite BaA. Description of data: The thermogravimetric (*TGA*) analysis to confirm the surface modification and to estimate the number of modifying molecules on the NPs surface. (PNG 21 kb)

Additional file 2: Figure S2. Interference of metabolic activity (MTT assay) with nanozeolites in a cell-free system. Interference of MTT dye with nanozeolites at concentrations range from 5 to 50 μg/mL (from 1.5 to 15 μg/cm^2^) in order to verify the credibility of the MTT assay in the cell-free system. (PDF 31 kb)

Additional file 3: Supplementary Material and Methods. (PDF 91 kb)

Additional file 4: Table S1. Statistical analysis of pairwise between groups comparison of nanozeolite internalization process. Description of data: Statistical analysis of the nanozeolite internalization data for the different types of nanozeolites in the same time point and for the same type nanozeolite in different time points. Data were evaluated by the Student *t* test. (PDF 10 kb)

Additional file 5: Table S2. Statistical analysis of pairwise between groups comparison of nanozeolite retention. Description of data: Statistical analysis of the nanozeolite retention data for the different types of nanozeolites in the same time point and for the same type nanozeolite in different time points. Data were evaluated by the Student *t* test. (PDF 51 kb)

## Abbreviations

[^133^Ba]Ba nanozeolite: nanozeolite A loaded with Ba^2+^ and spiked with ^133^Ba
BaA nanozeolite: nanozeolite A loaded with Ba^2+^
NaA nanozeolite: nanozeolite A loaded with Na^+^
NPs: nanoparticles
NR: neutral red assay
PEG: poly(ethylene) glycol
TEM: transmission electron microscopy
TGA: thermogravimetric analysis
