# Supplementary figures and images for: Effect of Surface Functionalization on the Cellular Uptake and Toxicity of Nanozeolite A
Source: Nanoscale Res Lett. 2016 Mar 2;11:123. doi: 10.1186/s11671-016-1334-8 (PMC4775514; doi:10.1186/s11671-016-1334-8)

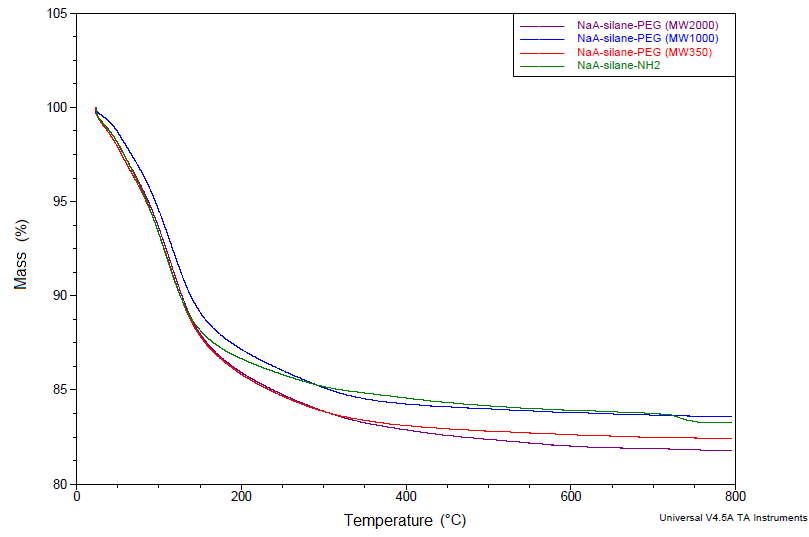

Supplement: Supplementary file 1 — TGA analysis of modified nanozeolite BaA. Description of data: The thermogravimetric (TGA) analysis to confirm the surface modification and to estimate the number of modifying molecules on the NPs surface. (PNG 21 kb) [file 11671_2016_1334_MOESM1_ESM.png]

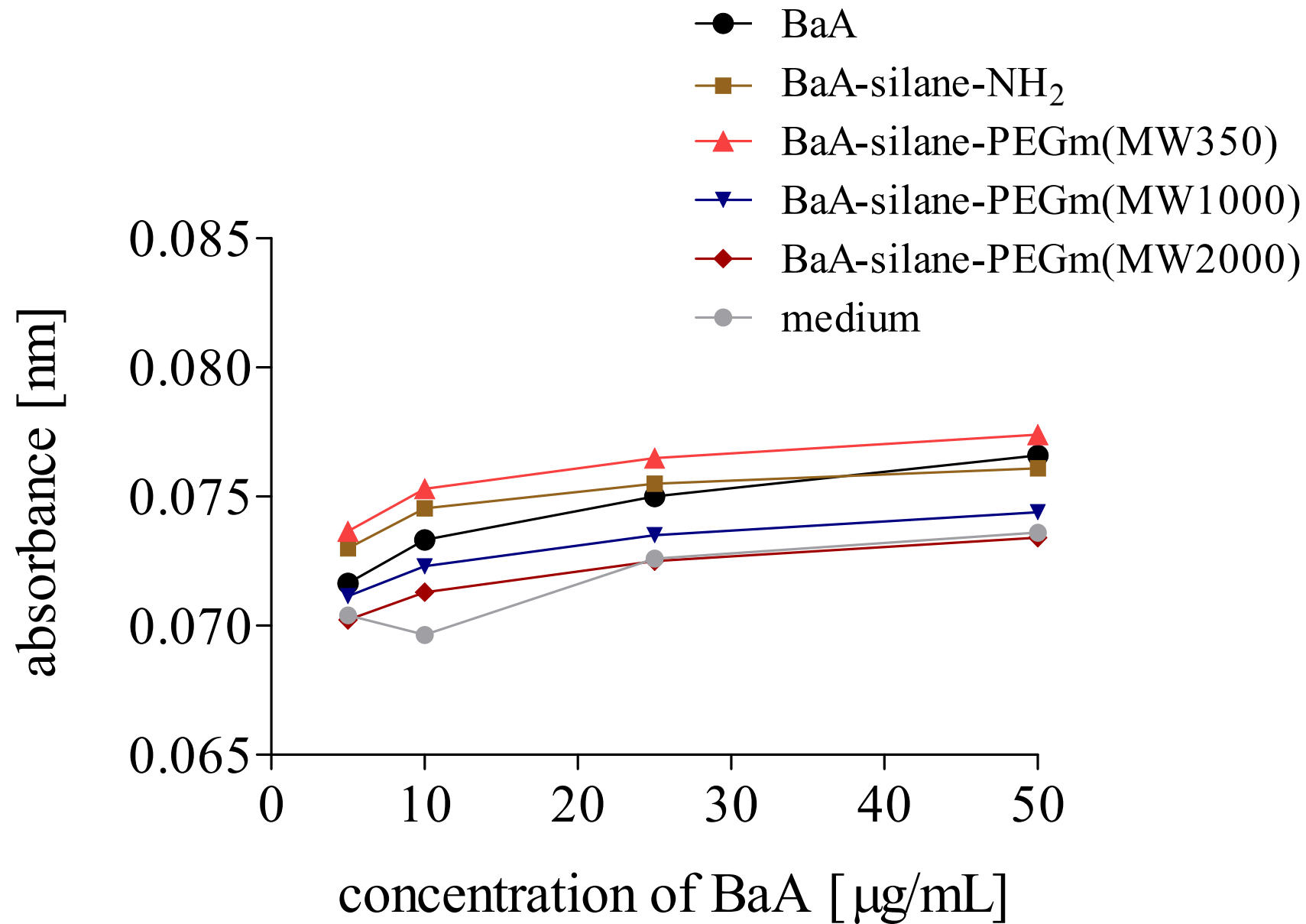

Supplement: Supplementary file 2 — Interference of metabolic activity (MTT assay) with nanozeolites in a cell-free system. Interference of MTT dye with nanozeolites at concentrations range from 5 to 50 μg/mL (from 1.5 to 15 μg/cm2) in order to verify the credibility of the MTT assay in the cell-free system. (PDF 31 kb) [file 11671_2016_1334_MOESM2_ESM.pdf]
