# Supplementary material for: Effect of Surface Functionalization on the Cellular Uptake and Toxicity of Nanozeolite A
Source: Nanoscale Res Lett. 2016 Mar 2;11:123. doi: 10.1186/s11671-016-1334-8 (PMC4775514; doi:10.1186/s11671-016-1334-8)
Supplement: Supplementary file 3 — Supplementary Material and Methods. (PDF 91 kb) [file 11671_2016_1334_MOESM3_ESM.pdf]

Supplementary Materials and Methods

Interference of metabolic activity (MTT assay) with nanozeolites in the cell free system.

A 100 µL aliquot of nanozeolites suspensions in the cell culture medium at concentrations of 5, 10, 25, 50 µg/mL (1.5, 3, 7.5, 15 µg/cm^2) were added to 96-well microplates (TPP, Switzerland) for 24 h. Next, 100 µL of 3 mg/mL MTT solution was added to each well and incubated at 37°C for 3 h. DMSO was added at a ratio of 3:1 to the MTT-nanozeolite mixture and incubated for another 10 min at room temperature. Absorbance was measured at 570 nm in plate reader spectrophotometer Infinite M200 (Tecan, Austria).
