# Supplementary material for: Effect of Surface Functionalization on the Cellular Uptake and Toxicity of Nanozeolite A
Source: Nanoscale Res Lett. 2016 Mar 2;11:123. doi: 10.1186/s11671-016-1334-8 (PMC4775514; doi:10.1186/s11671-016-1334-8)
Supplement: Supplementary file 5 — Statistical analysis of pairwise between groups comparison of nanozeolite retention. Description of data: Statistical analysis of the nanozeolite retention data for the different types of nanozeolites in the same time point and for the same type nanozeolite in different time points. Data were evaluated by the Student t test. (PDF 51 kb) [file 11671_2016_1334_MOESM5_ESM.pdf]

Supplementary Table 2. Statistical analysis of pairwise between groups comparison of nanozeolite retention.

Nanozeolite retention. Statistical significance of differences ($p<0.05$).			
Tme	3 h	6 h	24 h
3 h	**BaA vs BaAsiPEG1000,**<br>**BaA vs BaAsiPEG2000**<br>**BaAsiNH2 vs BaAsiPEG350**<br>**BaAsiNH2 vs BaAsiPEG1000**<br>**BaAsiNH2 vs BaAsiPEG2000**<br>**BaAsiPEG350 vs BaAsiPEG1000**<br>**BaAsiPEG350 vs BaAsiPEG2000**	*BaAsiNH2*<br>*BaAsiPEG2000*	*BaAsiNH2*<br>*BaAsiPEG2000*
6 h		**BaA vs BaAsiPEG350**<br>**BaA vs BaAsiPEG1000**<br>**BaA vs BaAsiPEG2000**<br>**BaAsiNH2 vs BaAsiPEG350**<br>**BaAsiNH2 vs BaAsiPEG1000**<br>**BaAsiNH2 vs BaAsiPEG2000**<br>**BaAsiPEG350 vs BaAsiPEG1000**<br>**BaAsiPEG350 vs BaAsiPEG2000**<br>**BaAsiPEG1000 vs BaAsiPEG2000**	*BaAsiPEG1000*
24 h			**BaA vs BaAsiNH2**<br>**BaA vs BaAsiPEG350**<br>**BaA vs BaAsiPEG1000**<br>**BaAsiNH2 vs BaAsiPEG1000**<br>**BaAsiNH2 vs BaAsiPEG2000**<br>**BaAsiPEG350 vs BaAsiPEG1000**<br>**BaAsiPEG350 vs BaAsiPEG2000**

The statistical significance of differences of the internalization data for the different types of nanozeolites in the same time point (show in bold typeface) was evaluated by the Student t-test for independent samples. The statistical significance of differences of the internalization data for the same type nanozeolite in different time points (show in italic typeface) was evaluated by the Student t-test for dependent samples.
